# Supplementary material for: A Two‐Component Pseudo‐Icosahedral Protein Nanocompartment with Variable Shell Composition and Irregular Tiling
Source: Adv Sci (Weinh). 2025 Jun 25;12(32):e03617. doi: 10.1002/advs.202503617 (PMC12407261; doi:10.1002/advs.202503617)
Supplement: Supplementary file 1 — Supporting Information [file ADVS-12-e03617-s001.pdf]

ADVANCED SCIENCE
Open Access

Supporting Information

for *Adv. Sci.*, DOI 10.1002/advs.202503617

A Two-Component Pseudo-Icosahedral Protein Nanocompartment with Variable Shell Composition and Irregular Tiling

*Cassandra A. Dutcher, Michael P. Andreas and Tobias W. Giessen**

Supporting Information

A two-component pseudo-icosahedral protein nanocompartment with variable shell composition and irregular tiling

Cassandra A. Dutcher[1], Michael P. Andreas[1], and Tobias W. Giessen[1*]

[1]Department of Biological Chemistry, University of Michigan Medical School, Ann Arbor, MI 48109, USA

*correspondence: tgiessen@umich.edu

Table of Contents

Fig. S1 Sequence alignment of Sl-Enc1 and Sl-Enc2	2
Fig. S2. Size-exclusion analysis of Sl-Enc1, Sl-Enc2 and mixed shells	3
Fig. S3. Cryo-EM micrographs of Sl-Enc1	4
Fig. S4. Analysis of Sl-Enc2 majority shells	5
Fig. S5. SDS-PAGE of Sl-Enc1 and Sl-Enc2 majority samples subjected to cryo-EM analysis	6
Fig. S6. Majority Sl-Enc1 cryo-EM data processing workflow	7
Fig. S7. Majority Sl-Enc2 cryo-EM data processing workflow	8
Fig. S8. Comparison of CBD orientations within Sl-Enc1 and Sl-Enc2	9
Fig. S9. Cryo-EM 3D classification analysis workflow for the 2-fold (pore) interaction	10
Fig. S10. Cryo-EM 3D classification analysis workflow for the 2-fold (P-domain) interaction	11
Fig. S11. Cryo-EM 3D classification analysis workflow for the 3-fold interaction	12
Fig. S12. Cryo-EM 3D classification analysis workflow for the 5-fold interaction	13
Fig. S13. Identified high quality classes for the 2-fold (pore) 3D classification analysis	14
Fig. S14. Identified high quality classes for the 2-fold (P-domain) 3D classification analysis	15
Fig. S15. Identified high quality classes for the 3-fold 3D classification analysis	16
Fig. S16. Identified high quality classes for the 5-fold 3D classification analysis	17
Fig. S17. Surface electrostatics of Sl-Enc1 2-fold interactions	18
Fig. S18. Cysteine mutant shell assembly and crosslinking mass spectrometry	19
Fig. S19. Location of the diagnostic motifs within the Sl-Enc1 and Sl-Enc2 protomers	20
Table S1. DNA sequences of gBlock Gene Fragments	21
Table S2. DNA sequences of PCR primers used to construct plasmids	24
Table S3. Primer pairs used to construct the plasmids used in this study	25
Table S4. Protein sequences used in this work	26
Table S5. Cryo-EM data collection, refinement, and validation statistics	28
References	29

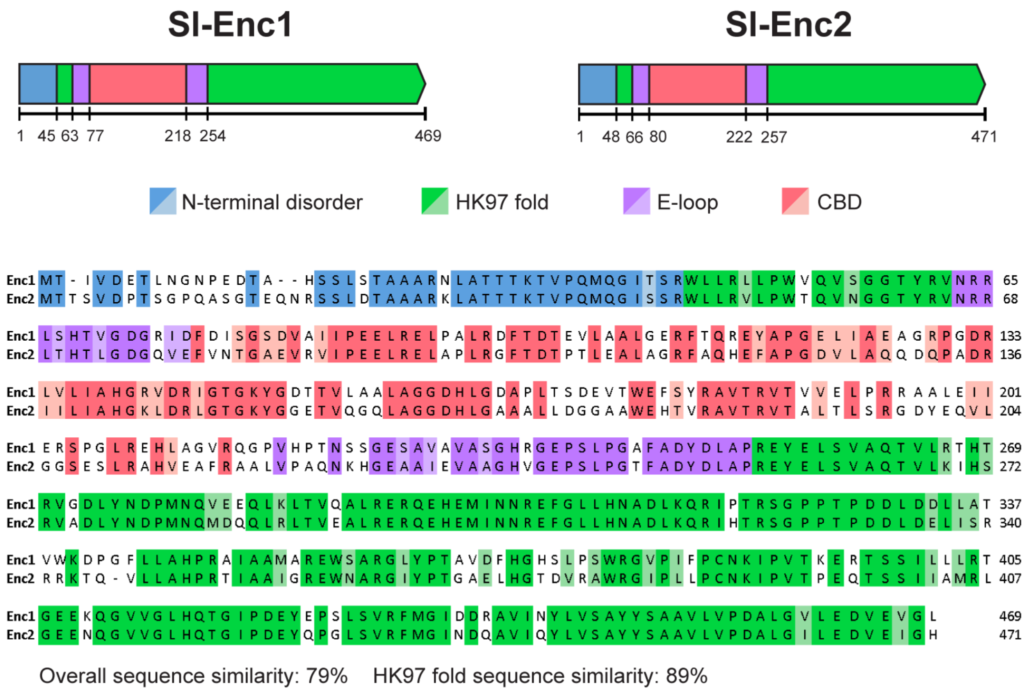

Fig. S1. Sequence alignment of Sl-Enc1 and Sl-Enc2. A sequence alignment of Sl-Enc1 and Sl-Enc2 generated via Clustal Omega is shown. Sequences are colored by domain.

A

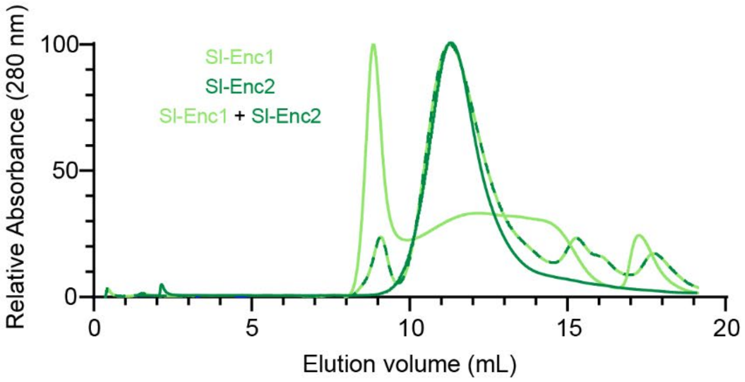

B

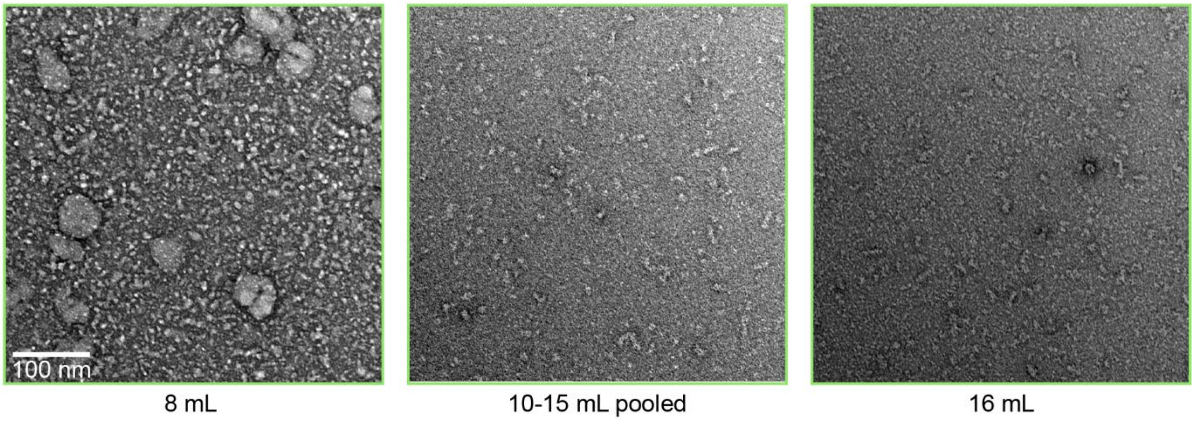

Fig. S2. Size-exclusion analysis of Sl-Enc1, Sl-Enc2 and mixed shells. (**A**) SEC chromatograms using a Superose 6 column. (**B**) Negative stain micrographs of the respective Sl-Enc1 elution volumes highlighting that no apparent encapsulin shells or other regular assembles can be observed.

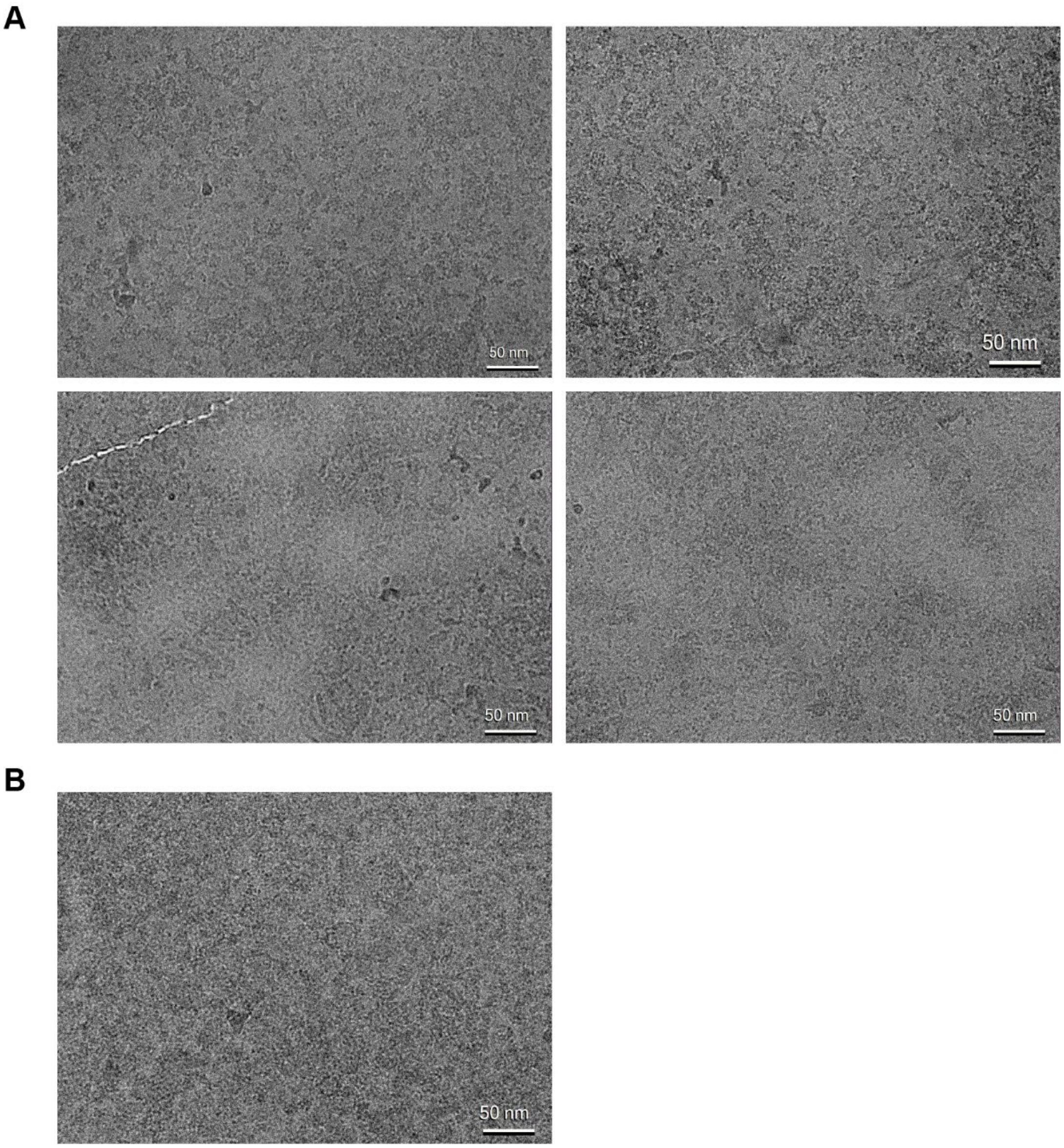

Fig. S3. Cryo-EM micrographs of Sl-Enc1. To prepare the Sl-Enc1 sample for vitrification, fractions between 10 and 15 mL of a Superose 6 run were pooled (see Fig. S1). (**A**) Representative micrographs of Sl-Enc1 in vitrified ice highlighting that no regular assemblies are present. (**B**) To exclude that any regular assemblies might stick to the grid carbon—and be excluded from the grid holes—we also imaged the carbon. No regular assemblies could be observed.

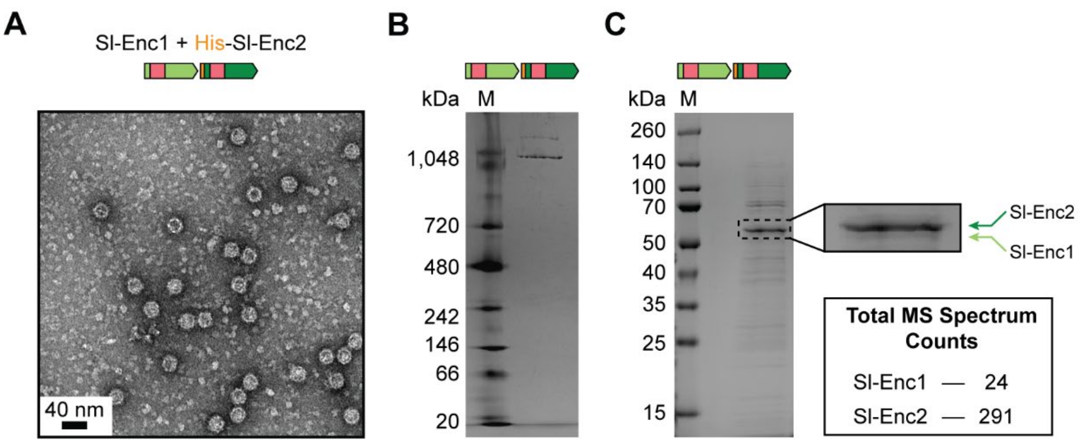

Fig. S4. Analysis of Sl-Enc2 majority shells. (**A**) Negative stain TEM micrograph of an Sl-Enc1 + His-Sl-Enc2 mixed shell sample purified via affinity purification and size exclusion chromatography. (**B**) Native PAGE analysis of an Sl-Enc1 majority mixed sample. M: molecular weight marker. (**C**) SDS-PAGE of purified Sl-Enc1 + His-Sl-Enc2. Outlined region on SDS-PAGE gel was excised and prepared for mass spectrometric analysis for protein identification. Total spectrum counts of Sl-Enc1 and Sl-Enc2 from mass spectrometry analysis are shown.

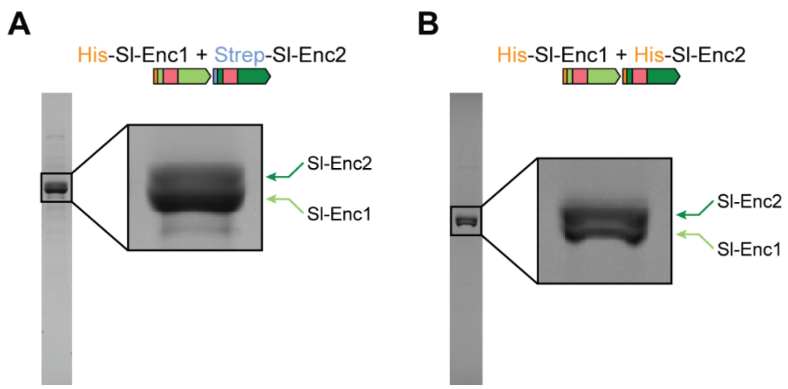

Fig. S5. SDS-PAGE analysis of Sl-Enc1 majority and Sl-Enc2 majority samples subjected to cryo-EM analysis. (**A**) SDS-PAGE gel lane of protein sample used for cryo-EM data collection resulting in a symmetry-averaged Sl-Enc1 shell map. (**B**) SDS-PAGE gel lane of protein sample used for cryo-EM data collection resulting in a symmetry-averaged Sl-Enc2 shell map.

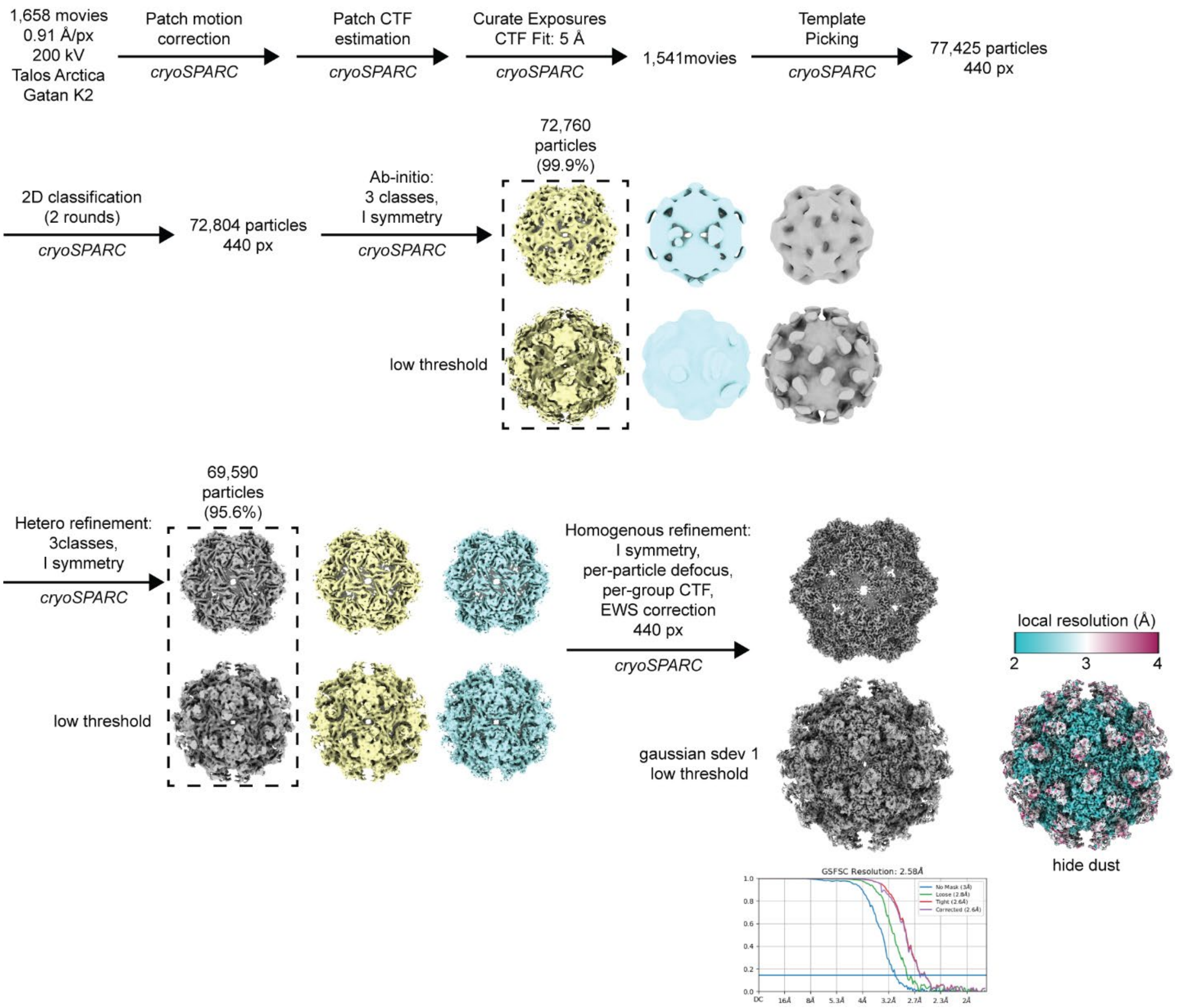

Fig. S6. Majority Sl-Enc1 cryo-EM data processing workflow. Cryo-EM workflow showing gold-standard Fourier shell correlation (FSC) curves for the final symmetry-averaged (I) consensus map as well as a local resolution analysis.

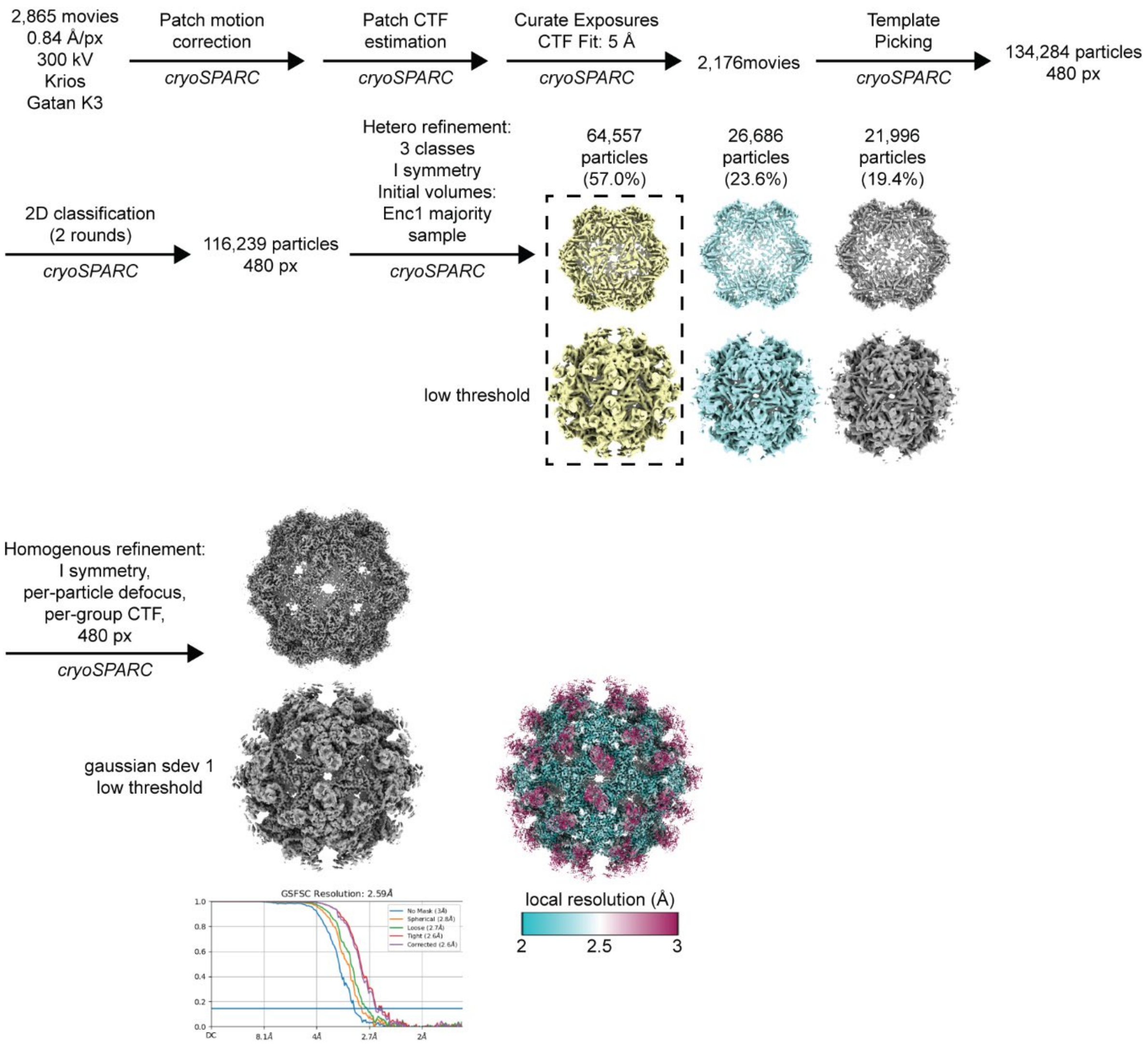

Fig. S7. Majority Sl-Enc2 cryo-EM data processing workflow. Cryo-EM workflow showing gold-standard Fourier shell correlation (FSC) curves for the final symmetry-averaged (I) consensus map as well as a local resolution analysis.

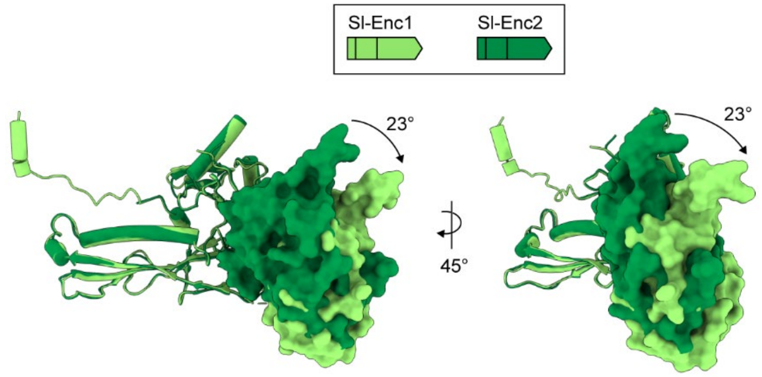

Fig. S8. Comparison of CBD orientations within Sl-Enc1 and Sl-Enc2. Structural alignment of the Sl-Enc1 and Sl-Enc2 proteins highlighting the different CBD tilts relative to the HK97-domain. A relative tilt between the Sl-Enc2 and Sl-Enc1 CBDs of ca. 23° can be observed. CBDs shown as surface, HK97-domain as ribbon.

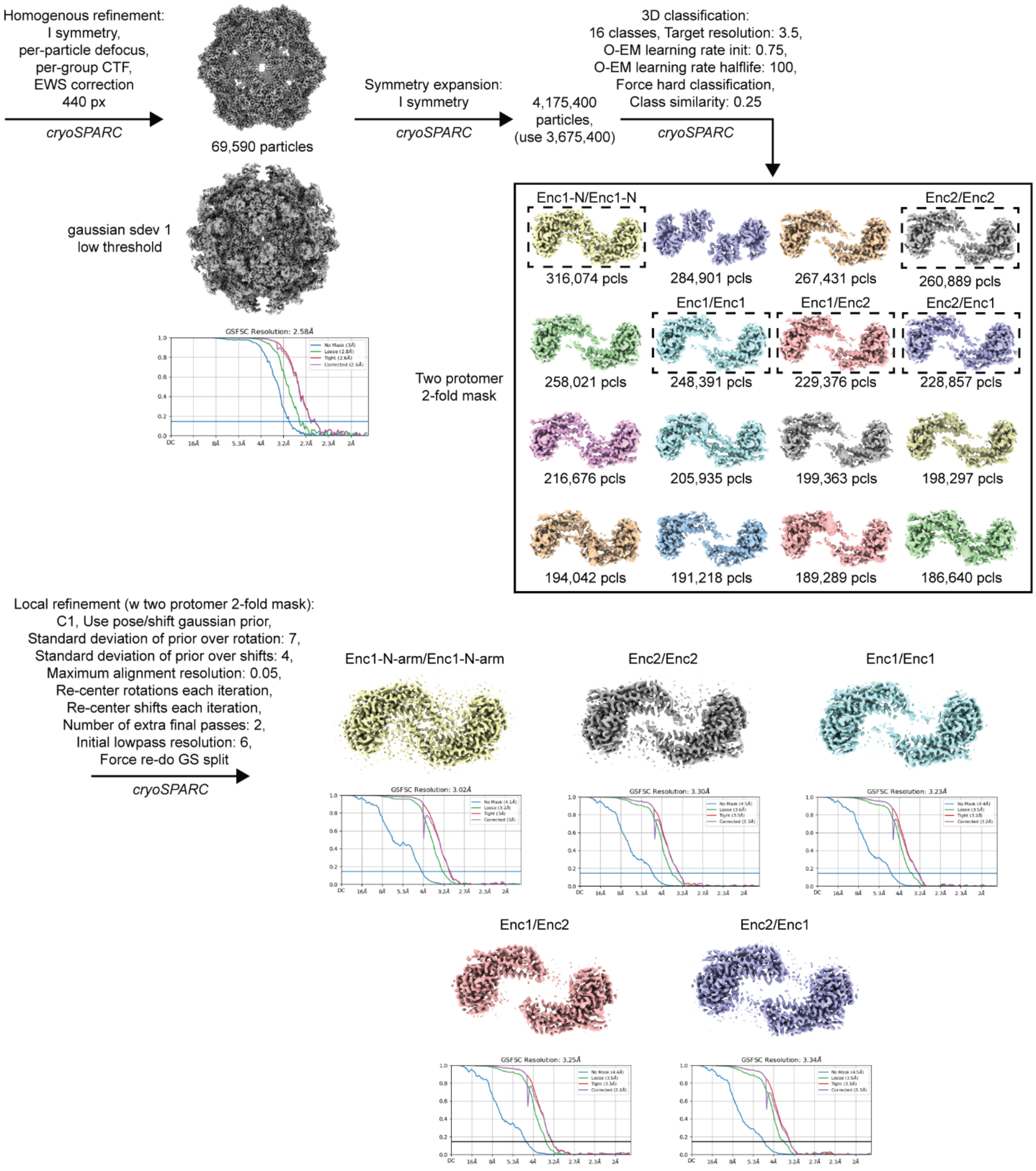

Fig. S9. Cryo-EM 3D classification analysis workflow for the 2-fold (pore) interaction.

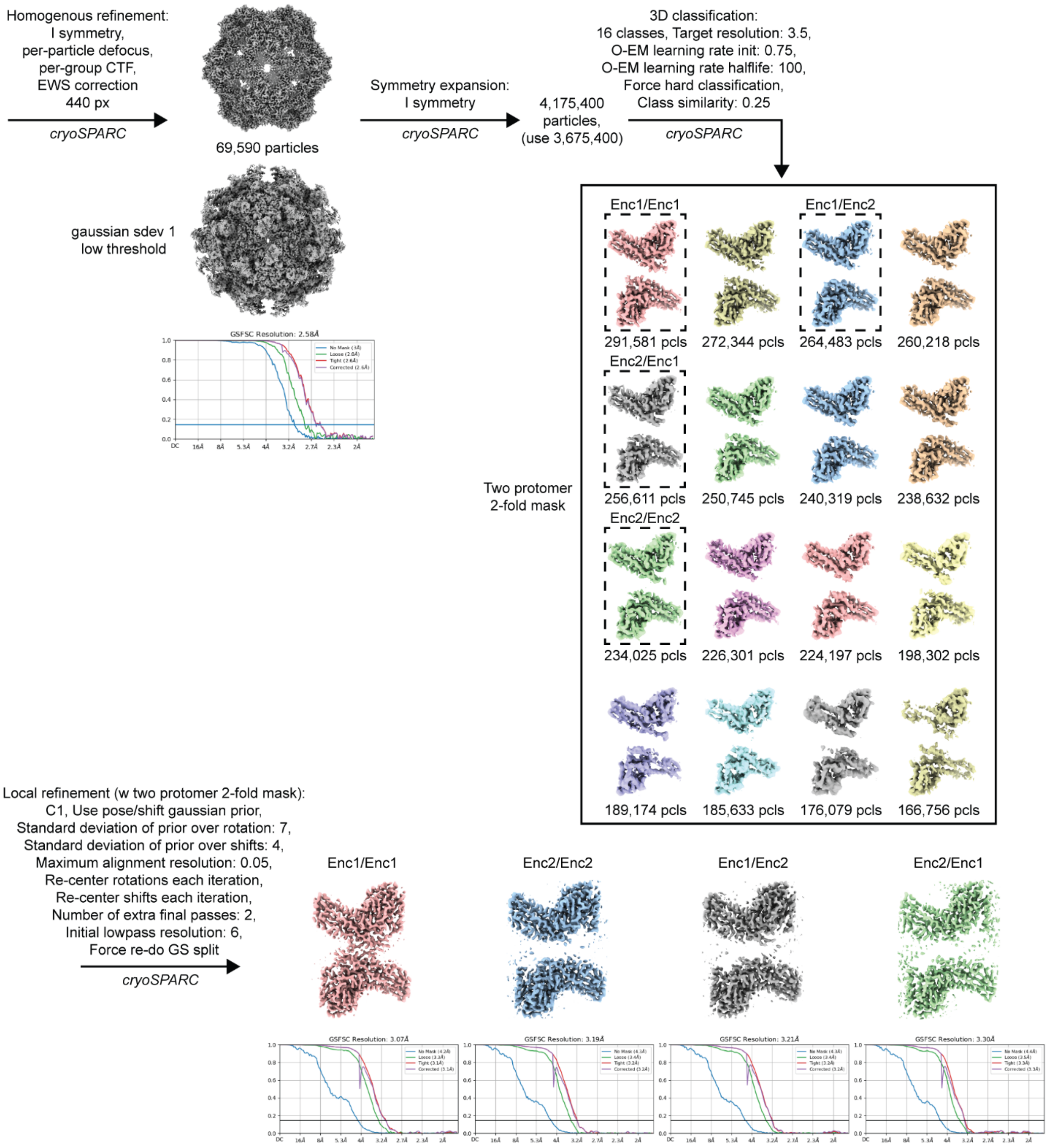

Fig. S10. Cryo-EM 3D classification analysis workflow for the 2-fold (P-domain) interaction.

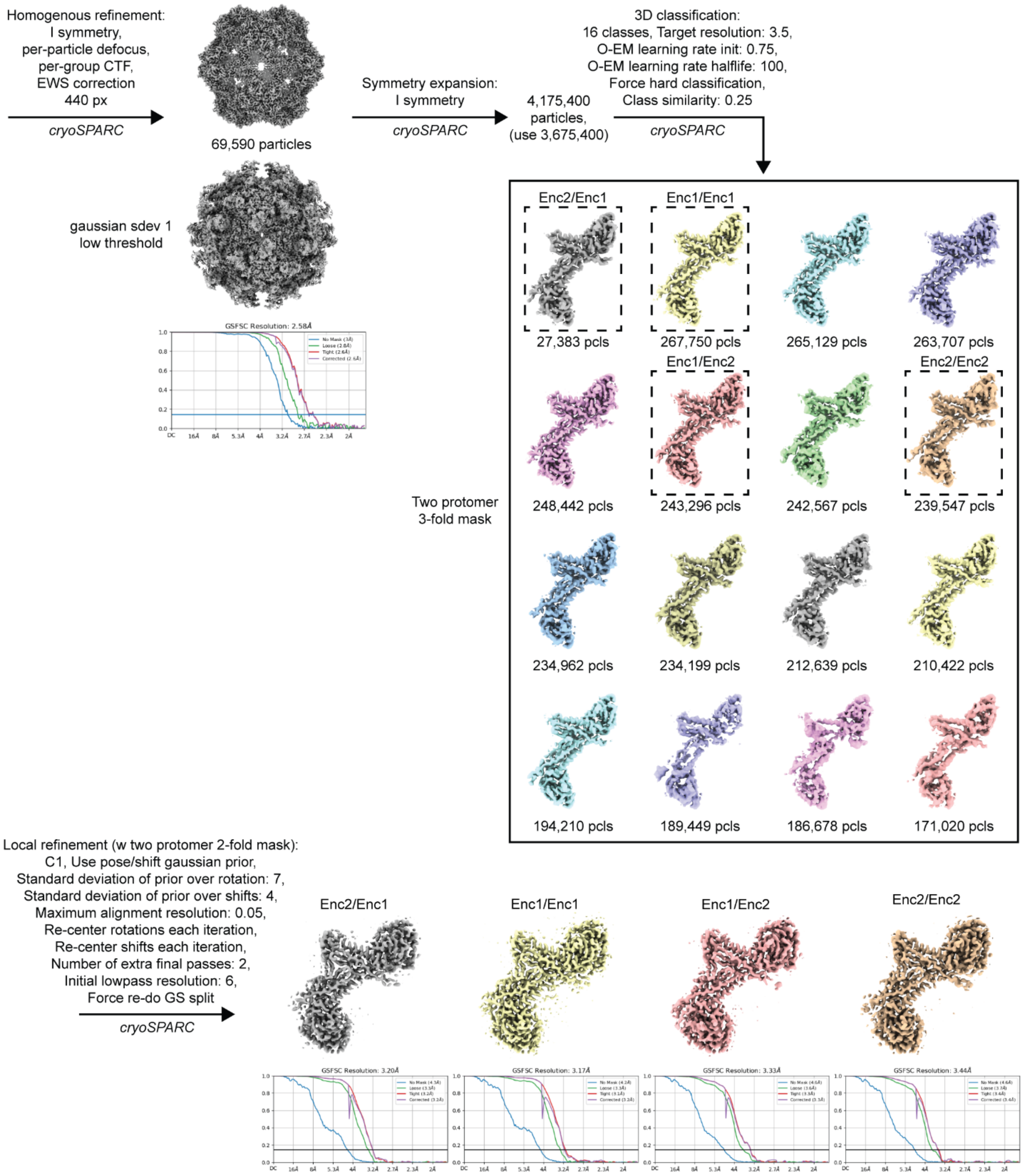

Fig. S11. Cryo-EM 3D classification analysis workflow for the 3-fold interaction.

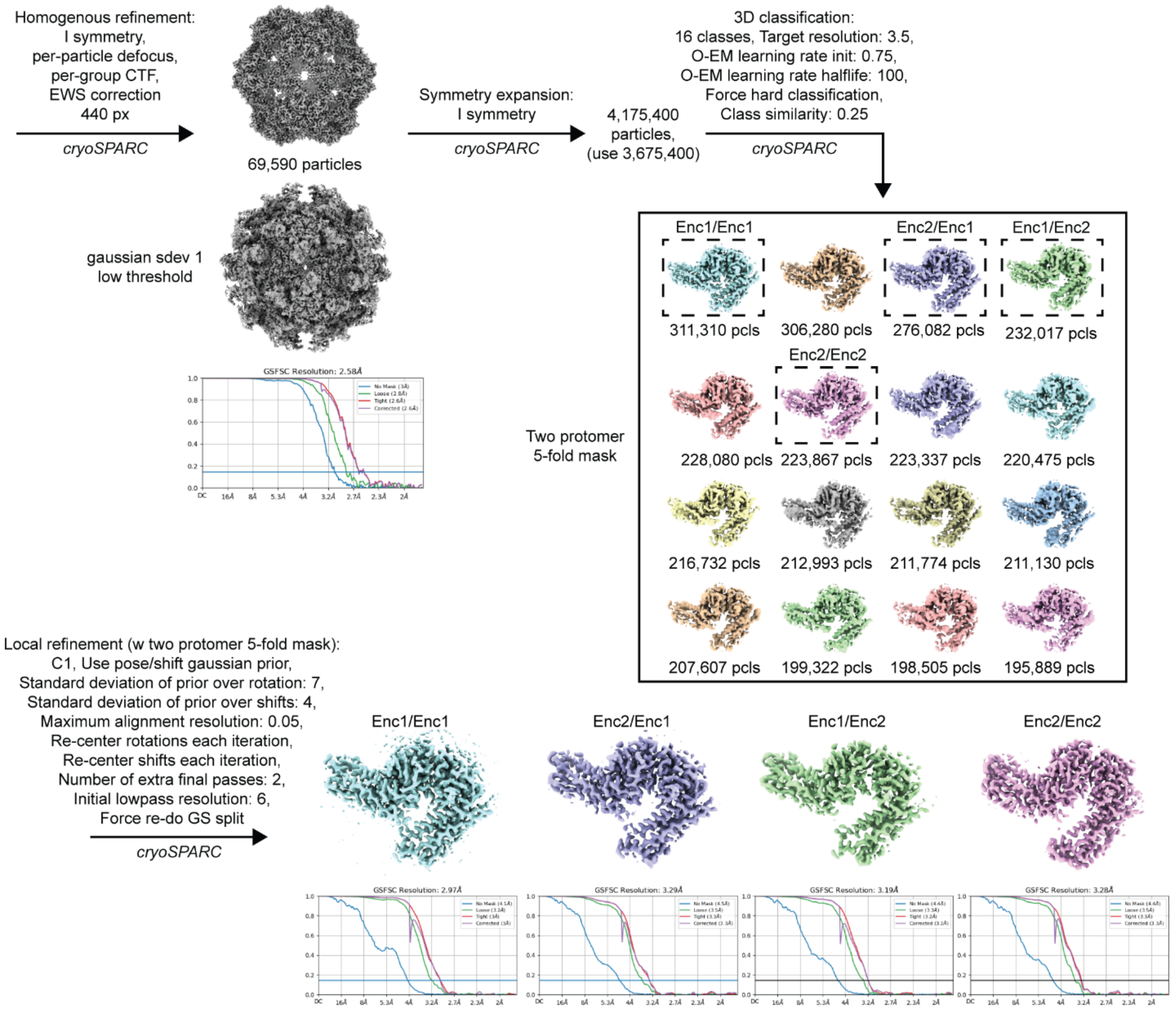

Fig. S12. Cryo-EM 3D classification analysis workflow for the 5-fold interaction.

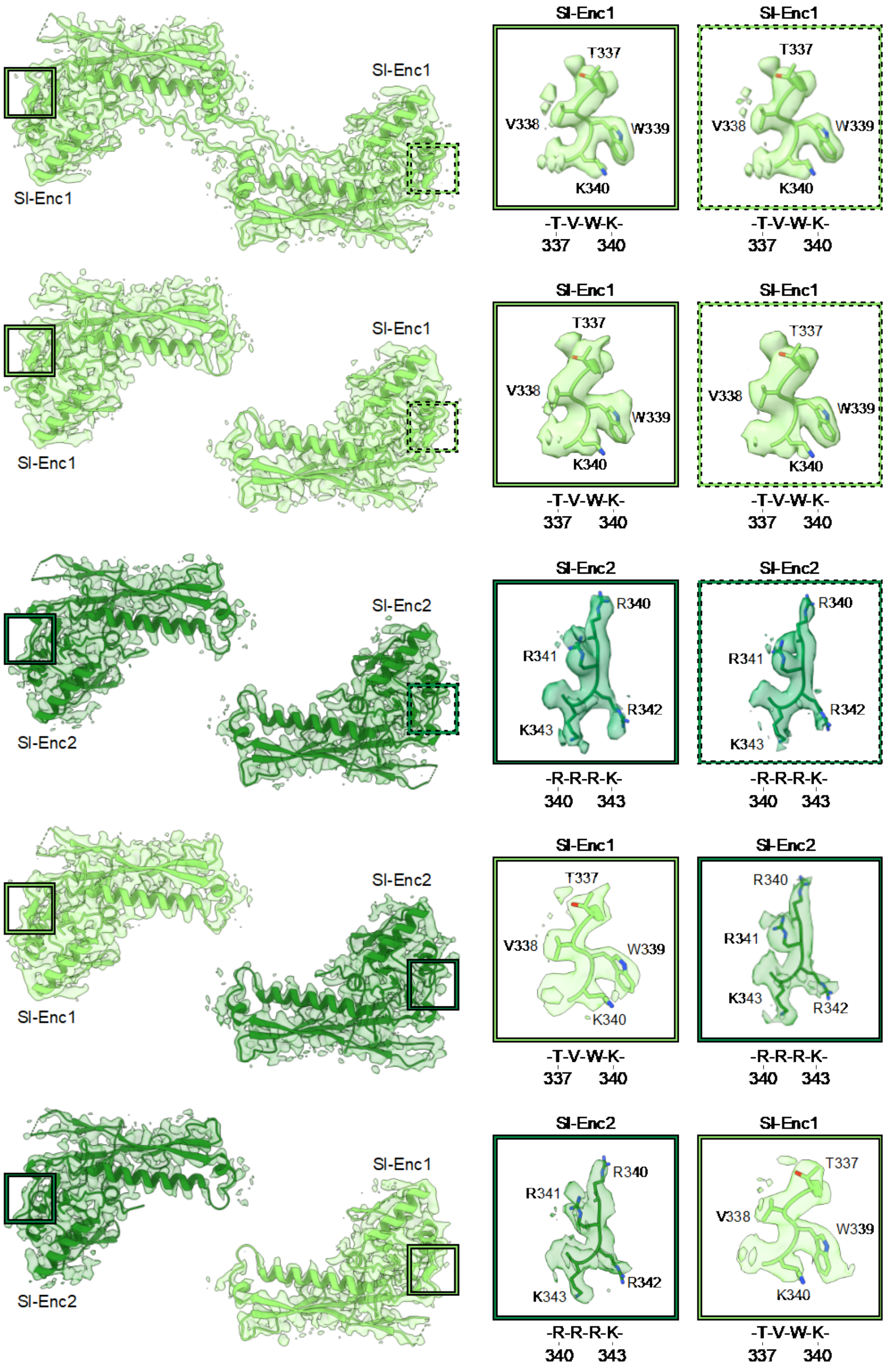

Fig. S13. Identified high quality classes for the 2-fold (pore) 3D classification analysis. Left: Cryo-EM densities and atomic models for the five identified classes representing all possible interaction permutations. The class shown on top represents the Sl-Enc1/Sl-Enc1 interaction with resolved N-arm. Right: Diagnostic regions located at the edge of the A-domain used to distinguish between Sl-Enc1 and Sl-Enc2.

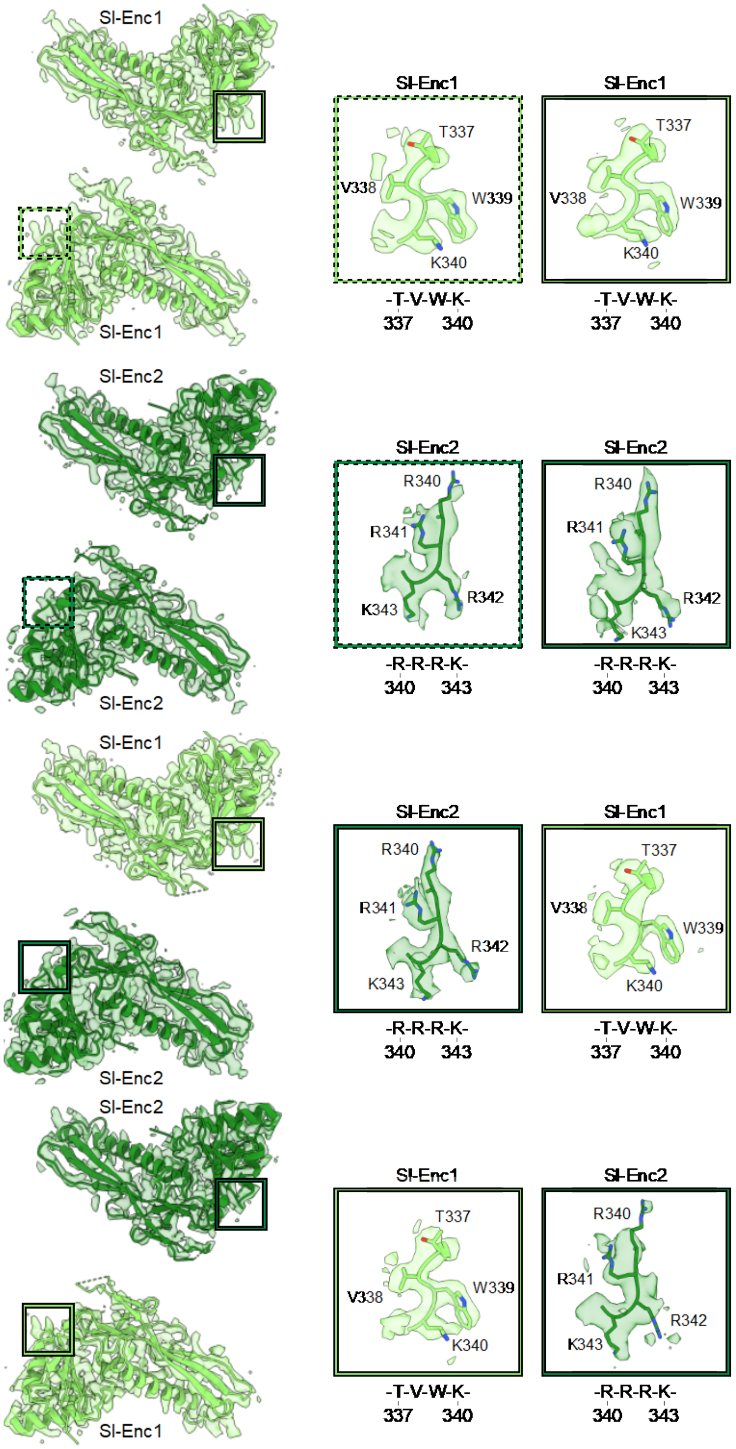

Fig. S14. Identified high quality classes for the 2-fold (P-domain) 3D classification analysis. Left: Cryo-EM densities and atomic models for the four identified classes representing all possible interaction permutations. Right: Diagnostic regions located at the edge of the A-domain used to distinguish between Sl-Enc1 and Sl-Enc2.

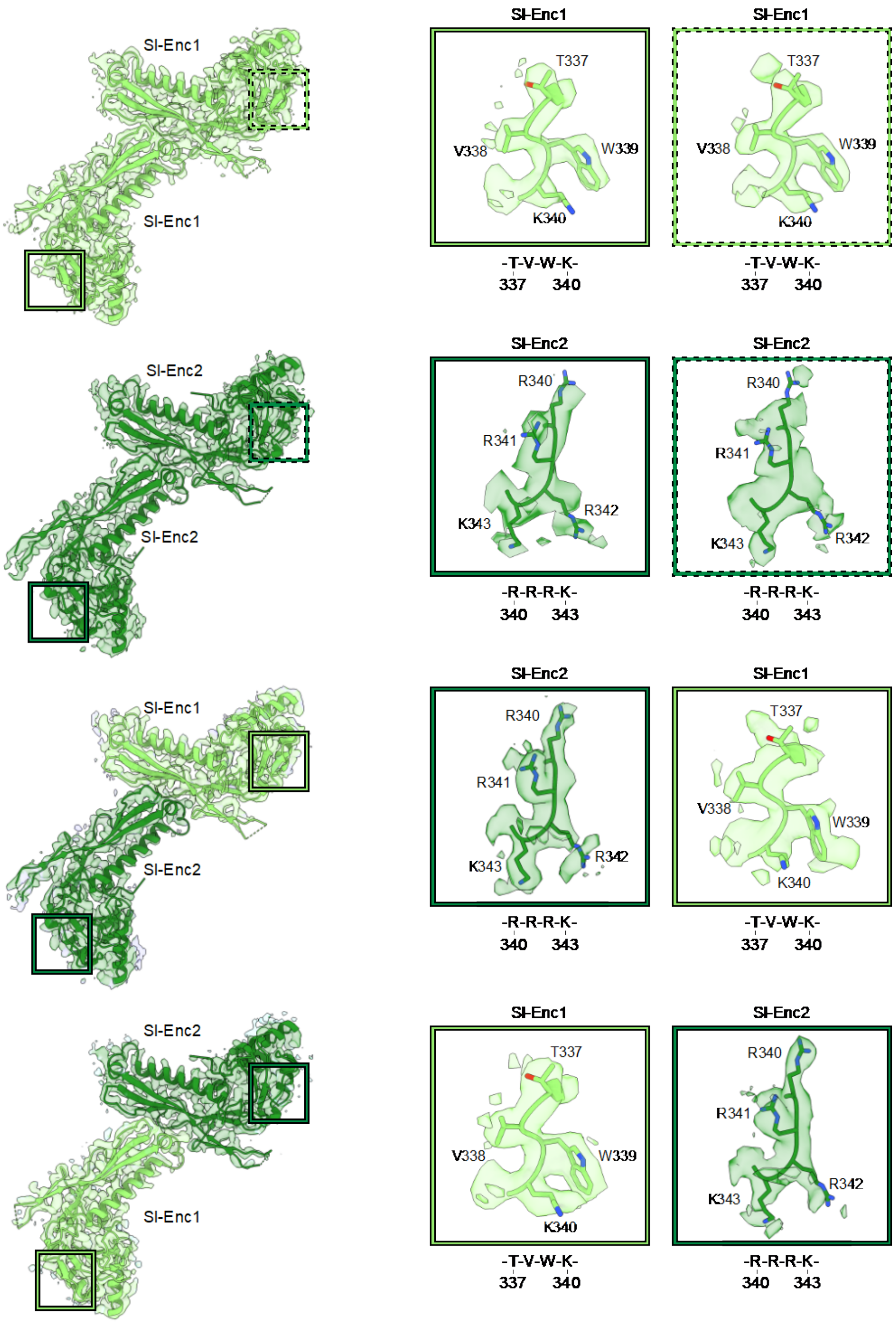

Fig. S15. Identified high quality classes for the 3-fold 3D classification analysis. Left: Cryo-EM densities and atomic models for the four identified classes representing all possible interaction permutations. Right: Diagnostic regions located at the edge of the A-domain used to distinguish between Sl-Enc1 and Sl-Enc2.

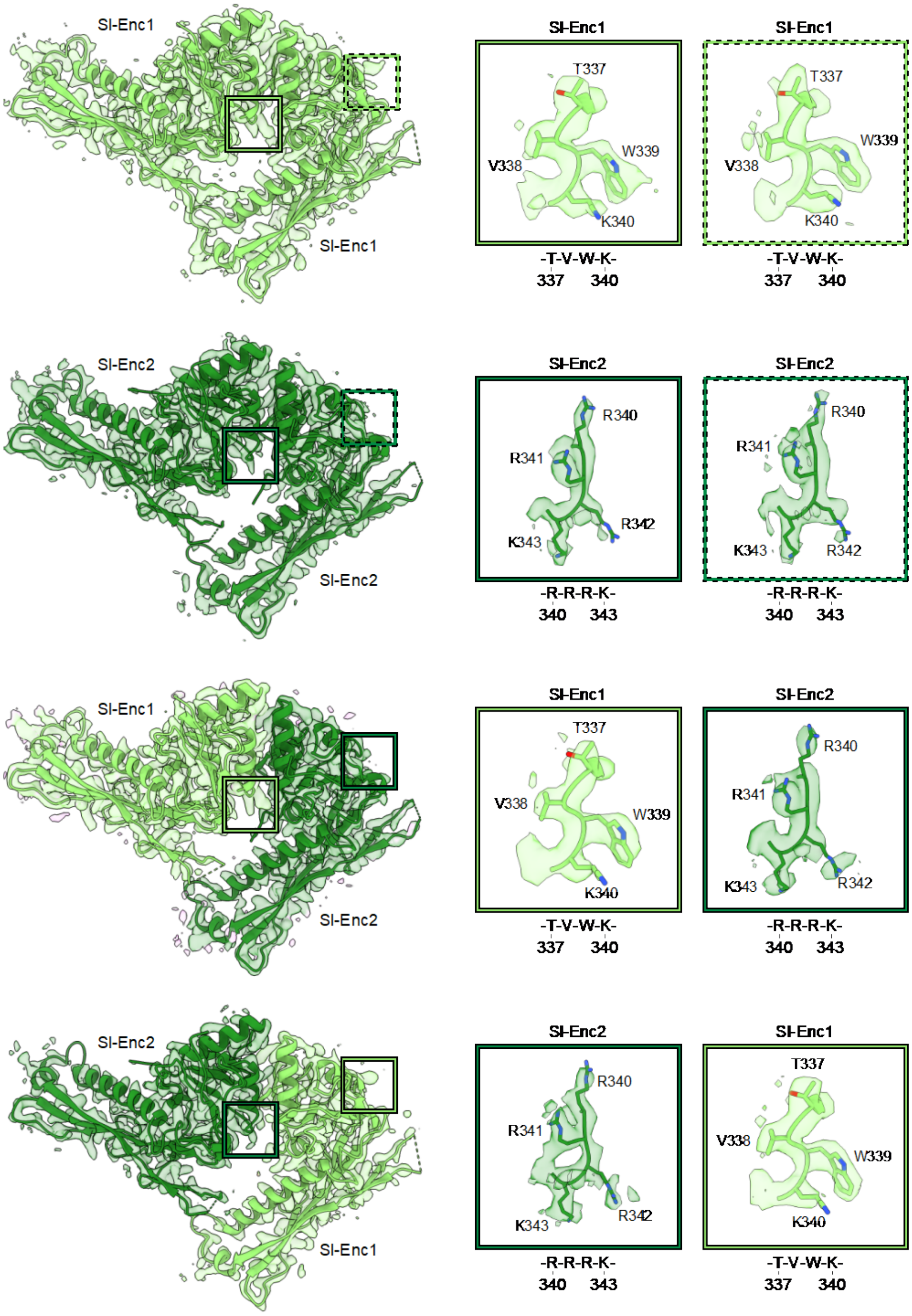

Fig. S16. Identified high quality classes for the 5-fold 3D classification analysis. Left: Cryo-EM densities and atomic models for the four identified classes representing all possible interaction permutations. Right: Diagnostic regions located at the edge of the A-domain used to distinguish between Sl-Enc1 and Sl-Enc2.

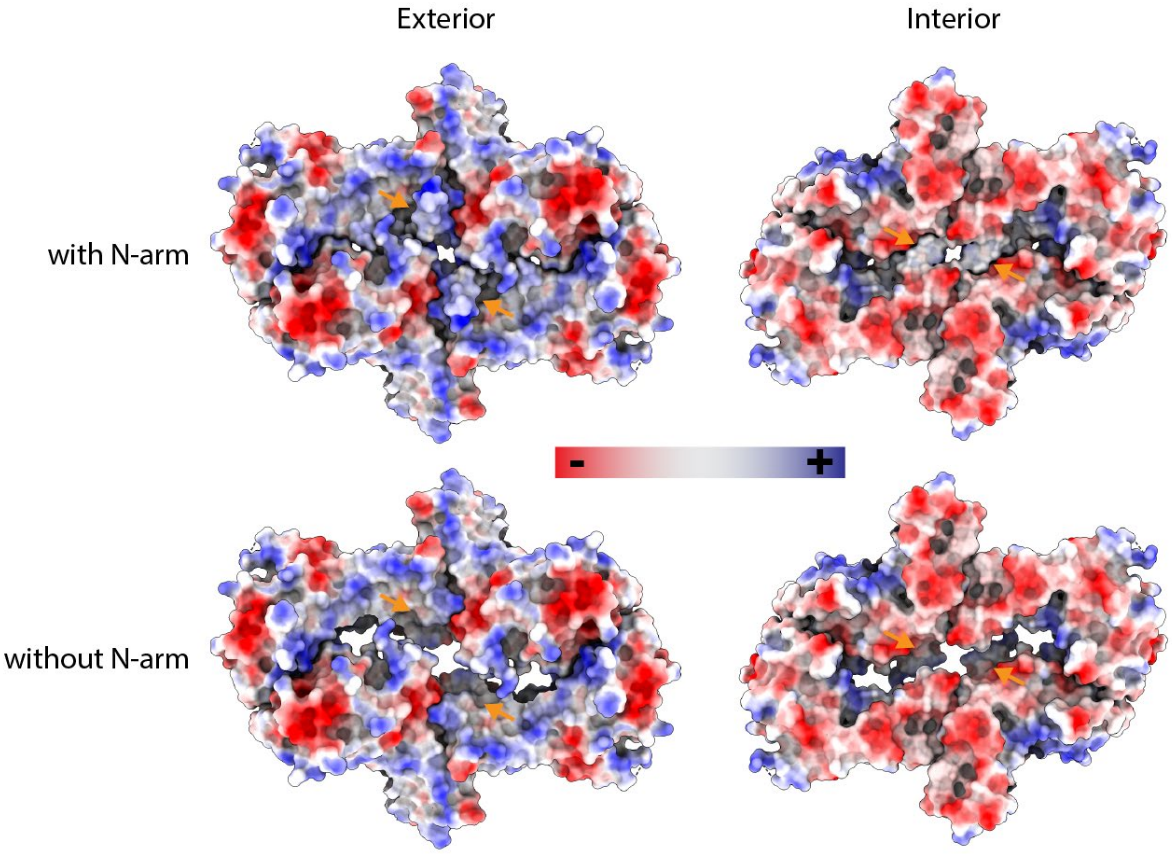

Fig. S17. Surface electrostatics of Sl-Enc1 2-fold interactions. The presence of two N-arms results in a smaller 2-fold pore and a difference in surface charge surrounding the pore (top) as compared to a 2-fold pore without N-arms present (bottom). Differences are highlighted with orange arrows.

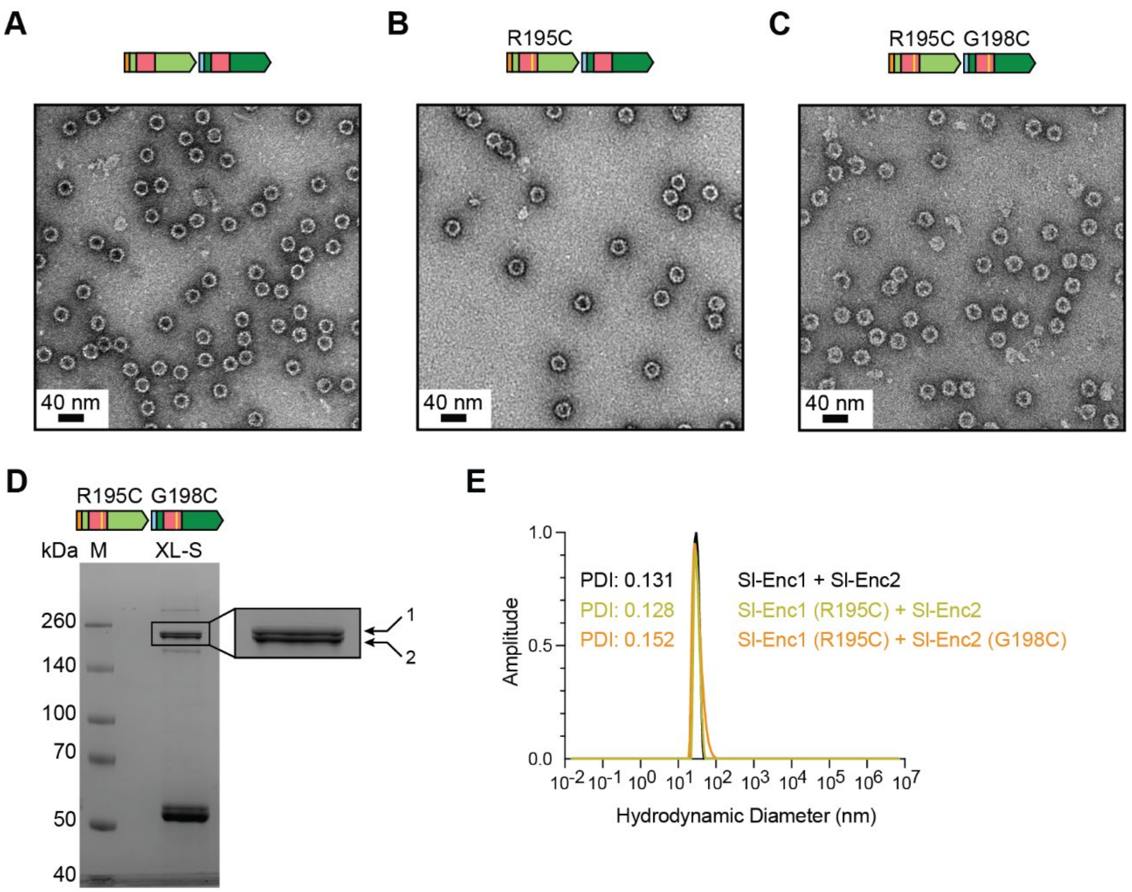

Fig. S18. Cysteine mutant shell assembly and crosslinking mass spectrometry. (**A**) Negative stain TEM micrograph of a mixed shell sample containing His-Sl-Enc1 and Strep-Sl-Enc2. (**B**) Negative stain TEM micrograph of a mixed shell sample containing mutant His-Sl-Enc1(R195C) and Strep-Sl-Enc2. (**C**) Negative stain TEM micrograph of a mixed shell sample containing mutant His-Sl-Enc1(R195C) and mutant Strep-Sl-Enc2 (G198C). (**D**) SDS-PAGE analysis of crosslinked double-cysteine mutant sample used for mass spectrometric analysis. M: molecular weight marker. XL-S: Crosslinked His-Sl-Enc1(R195C) + Strep-Sl-Enc2 (G198C) shells. (**E**) Comparative DLS measurements of wild-type and mutated mixed shells. PDI: polydispersity index.

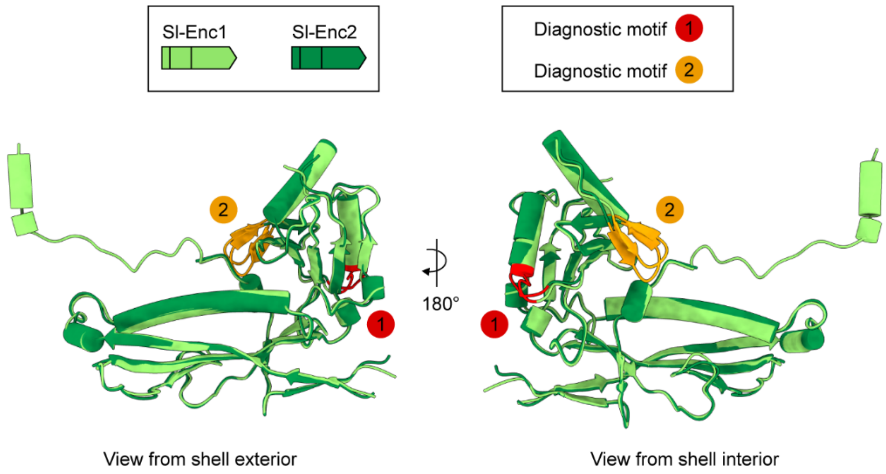

Fig. S19. Location of the diagnostic motifs within the Sl-Enc1 and Sl-Enc2 protomers. The two conserved differences between Enc1 and Enc2 shell proteins are highlighted. CBDs not shown for visual clarity.

Table S1. DNA sequences of gBlock Gene Fragments.

gBlock	DNA Sequence
SI-Enc1	AAGTATAAGAAGGAGATATACAATGACAATCGTAGACGAGACGTTAAATGG<br>GAACCCAGAGGACACCGCCCATTCGTCTTTGTCTACGGCTGCCGCCCGCA<br>ACTTGGCCACCACAACCAAAACTGTACCGCAAATGCAGGGCATCACATCC<br>CGTTGGTTGCTTCGTTTGTTACCGTGGGTTCAGGTCTCAGGGGGCACGTA<br>CCGCGTAAACCGCCGTTTATCACACACCGTGGGGGATGGACGTATCGACT<br>TTGATATTTCTGGTAGTGACGTAGCGATTATCCCCGAAGAATTACGCGAAC<br>TTCCAGCGCTTCGCGACTTCACGGATACTGAGGTCCTTGCTGCATTGGGG<br>GAGCGTTTCACTCAGCGTGAGTATGCCCCAGGAGAGTTGATCGCAGAGGC<br>TGGGCGCCCAGGGGATCGCCTTGTTTTAATCGCGCATGGCCGCGTAGATC<br>GCATTGGAACTGGAAAGTACGGCGACACCACCGTACTTGCAGCGCTTGCG<br>GGGGGCGACCACCTGGGGGACGCGCCGCTTACCTCTGACGAAGTTACAT<br>GGGAATTTTCGTATCGCGCGGTTACTCGCGTAACAGTAGTCGAGCTTCCG<br>CGCCGCGCCGCCTTAGAAATCATCGAACGTTCCCCAGGTCTTCGTGAGCA<br>TTTAGCCGGGGTACGTCAAGGGCCGGTTCACCCCACAAACAGTTCCGGCG<br>AATCTGCTGTAGCCGTGGCTAGTGGTCACCGTGGCGAGCCGTCGCTGCC<br>CGGAGCGTTCGCGGATTATGACTTAGCGCCCCGCGAGTACGAACTTAGCG<br>TCGCTCAGACAGTATTGCGCACGCATACGCGCGTGGGGGATTTATACAAT<br>GATCCTATGAACCAGGTGGAAGAACAATTAAAATTGACCGTCCAGGCACTG<br>CGCGAACGCCAGGAACATGAGATGATCAACAATCGTGAGTTCGGATTGCT<br>TCACAATGCAGACTTAAAGCAACGTATTCCTACGCGTAGCGGACCGCCTA<br>CTCCTGATGACTTGGATGATTTGCTGGCGACAGTTTGGAAGGACCCTGGC<br>TTTCTTTTAGCGCATCCACGCGCCATCGCAGCAATGGCACGTGAATGGTC<br>GGCTCGCGGACTGTATCCAACGGCGGTCGATTTTCATGGGCATTCTCTTC<br>CATCCTGGCGTGGTGTTCCAATCTTTCCGTGCAATAAAATTCCAGTCACTA<br>AGGAACGCACAAGCTCCATTCTGCTTCTTCGCACCGGCGAGGAAAAGCAG<br>GGCGTCGTCGGCCTTCACCAAACTGGGATTCCTGACGAGTATGAGCCAAG<br>TCTGTCAGTTCGTTTCATGGGAATCGATGACCGCGCTGTAATTAACTACTT<br>GGTTTCTGCGTACTACAGTGCAGCCGTGCTTGTACCAGATGCATTGGGAG<br>TCTTAGAGGATGTCGAAGTCGGGTTATGAATTAACCTAGGCTGCTGC
SI-Enc2	AAGTATAAGAAGGAGATATACAATGACTACGTCCGTGGATCCAACTTCAGG<br>TCCTCAGGCAAGTGGGACAGAGCAGAATCGCTCAAGCTTGGACACAGCCG<br>CTGCTCGTAAACTGGCTACTACCACGAAAACGGTCCCGCAAATGCAGGGC<br>ATTTCATCACGCTGGTTGCTTCGTGTCTTACCGTGGACGCAGGTCAATGG<br>GGGTACATACCGTGTTAATCGCCGCCTGACTCATACCCTGGGAGACGGTC<br>AAGTGGAGTTCGTAAACACCGGCGCTGAAGTTCGTGTAATTCCGGAGGAG<br>CTGCGTGAGTTGGCCCCGCTTCGTGGGTTTACCGATACTCCAACTCTGGA<br>AGCCCTTGCGGGTCGCTTTGCACAACACGAGTTTGCTCCAGGCGATGTCT<br>TAGCTCAGCAGGACCAACCGGCTGACCGCATCATCTTGATTGCTCACGGT<br>AAGTTGGATCGTTTAGGCACTGGCAAGTATGGCGGAGAAACGGTGCAAGG<br>GCAGTTGGCCGGCGGTGACCACCTGGGAGCGGCCGCACTGTTAGACGGT

	GGTGCGGCCTGGGAGCATACTGTCCGCGCTGTTACCCGCGTCACTGCAC<br>TGACTTTATCGCGCGGTGATTATGAACAAGTCCTGGGTGGTTCCGAGAGC<br>TTACGTGCGCACGTCGAAGCTTTTCGTGCGGCCTTAGTACCGGCTCAGAA<br>TAAGCACGGCGAGGCTGCGATCGAAGTAGCTGCCGGTCATGTCGGGGAA<br>CCCTCCCTGCCAGGGACGTTTGCCGATTACGATCTTGCTCCTCGTGAGTA<br>CGAGCTTTCAGTAGCCCAGACGGTTTTAAAGATTCACTCTCGTGTTGCCGA<br>CCTGTATAATGACCCCATGAACCAAATGGATCAGCAATTACGCTTAACTGT<br>CGAGGCTTTGCGCGAACGCCAAGAACATGAGATGATCAACAACCGTGAAT<br>TTGGTTTACTGCATAATGCAGATTTAAAGCAGCGCATCCATACGCGCTCCG<br>GCCCCCCAACACCTGACGACCTGGACGAGTTAATCTCTCGCCGCCGCAAA<br>ACGCAAGTGTTGTTGGCGCATCCACGCACTATTGCCGCCATTGGTCGTGA<br>GTGGAACGCGCGTGGAATCTATCCGACGGGCGCCGAATTGCATGGAACA<br>GATGTACGCGCTTGGCGTGGAATTCCTTTACTTCCTTGTAACAAAATTCCC<br>GTAACCCCGGAACAAACAAGCAGTATCATTGCTATGCGCTTGGGTGAGGA<br>AAACCAGGGGGTAGTCGGATTGCACCAGACCGGCATTCCTGACGAGTATC<br>AACCAGGCTTGTCTGTTCGTTTTATGGGGATTAATGATCAGGCAGTCATCC<br>AGTATCTTGTTAGCGCGTACTATTCAGCAGCAGTGTTGGTGCCTGATGCTT<br>TGGGGATTCTGGAGGATGTTGAAATCGGGCATTGAATTAACCTAGGCTGC<br>TGC
SI-<br>Enc1+SI-<br>Enc2	AAGTATAAGAAGGAGATATACAATGACAATCGTAGACGAGACGTTAAATGG<br>GAACCCAGAGGACACCGCCCATTCGTCTTTGTCTACGGCTGCCGCCCGCA<br>ACTTGGCCACCACAACCAAAACTGTACCGCAAATGCAGGGCATCACATCC<br>CGTTGGTTGCTTCGTTTGTTACCGTGGGTTCAGGTCTCAGGGGGCACGTA<br>CCGCGTAAACCGCCGTTTATCACACACCGTGGGGGATGGACGTATCGACT<br>TTGATATTTCTGGTAGTGACGTAGCGATTATCCCCGAAGAATTACGCGAAC<br>TTCCAGCGCTTCGCGACTTCACGGATACTGAGGTCCTTGCTGCATTGGGG<br>GAGCGTTTCACTCAGCGTGAGTATGCCCCAGGAGAGTTGATCGCAGAGGC<br>TGGGCGCCCAGGGGATCGCCTTGTTTTAATCGCGCATGGCCGCGTAGATC<br>GCATTGGAACTGGAAAGTACGGCGACACCACCGTACTTGCAGCGCTTGCG<br>GGGGGCGACCACCTGGGGGACGCGCCGCTTACCTCTGACGAAGTTACAT<br>GGGAATTTTCGTATCGCGCGGTTACTCGCGTAACAGTAGTCGAGCTTCCG<br>CGCCGCGCCGCCTTAGAAATCATCGAACGTTCCCCAGGTCTTCGTGAGCA<br>TTTAGCCGGGGTACGTCAAGGGCCGGTTCACCCCACAAACAGTTCCGGCG<br>AATCTGCTGTAGCCGTGGCTAGTGGTCACCGTGGCGAGCCGTCGCTGCC<br>CGGAGCGTTCGCGGATTATGACTTAGCGCCCCGCGAGTACGAACTTAGCG<br>TCGCTCAGACAGTATTGCGCACGCATACGCGCGTGGGGGATTTATACAAT<br>GATCCTATGAACCAGGTGGAAGAACAATTAAAATTGACCGTCCAGGCACTG<br>CGCGAACGCCAGGAACATGAGATGATCAACAATCGTGAGTTCGGATTGCT<br>TCACAATGCAGACTTAAAGCAACGTATTCCTACGCGTAGCGGACCGCCTA<br>CTCCTGATGACTTGGATGATTTGCTGGCGACAGTTTGGAAGGACCCTGGC<br>TTTCTTTTAGCGCATCCACGCGCCATCGCAGCAATGGCACGTGAATGGTC<br>GGCTCGCGGACTGTATCCAACGGCGGTCGATTTTCATGGGCATTCTCTTC<br>CATCCTGGCGTGGTGTTCCAATCTTTCCGTGCAATAAAATTCCAGTCACTA

	AGGAACGCACAAGCTCCATTCTGCTTCTTCGCACCGGCGAGGAAAAGCAG<br>GGCGTCGTCGGCCTTCACCAAACTGGGATTCCTGACGAGTATGAGCCAAG<br>TCTGTCAGTTCGTTTCATGGGAATCGATGACCGCGCTGTAATTAACTACTT<br>GGTTTCTGCGTACTACAGTGCAGCCGTGCTTGTACCAGATGCATTGGGAG<br>TCTTAGAGGATGTCGAAGTCGGGTTATGATATAAGAAGGAGATATACATGA<br>CTACGTCCGTGGATCCAACTTCAGGTCCTCAGGCAAGTGGGACAGAGCAG<br>AATCGCTCAAGCTTGGACACAGCCGCTGCTCGTAAACTGGCTACTACCAC<br>GAAAACGGTCCCGCAAATGCAGGGCATTTCATCACGCTGGTTGCTTCGTG<br>TCTTACCGTGGACGCAGGTCAATGGGGGTACATACCGTGTTAATCGCCGC<br>CTGACTCATACCCTGGGAGACGGTCAAGTGGAGTTCGTAAACACCGGCGC<br>TGAAGTTCGTGTAATTCCGGAGGAGCTGCGTGAGTTGGCCCCGCTTCGTG<br>GGTTTACCGATACTCCAACTCTGGAAGCCCTTGCGGGTCGCTTTGCACAA<br>CACGAGTTTGCTCCAGGCGATGTCTTAGCTCAGCAGGACCAACCGGCTGA<br>CCGCATCATCTTGATTGCTCACGGTAAGTTGGATCGTTTAGGCACTGGCAA<br>GTATGGCGGAGAAACGGTGCAAGGGCAGTTGGCCGGCGGTGACCACCTG<br>GGAGCGGCCGCACTGTTAGACGGTGGTGCGGCCTGGGAGCATACTGTCC<br>GCGCTGTTACCCGCGTCACTGCACTGACTTTATCGCGCGGTGATTATGAA<br>CAAGTCCTGGGTGGTTCCGAGAGCTTACGTGCGCACGTCGAAGCTTTTCG<br>TGCGGCCTTAGTACCGGCTCAGAATAAGCACGGCGAGGCTGCGATCGAA<br>GTAGCTGCCGGTCATGTCGGGGAACCCTCCCTGCCAGGGACGTTTGCCG<br>ATTACGATCTTGCTCCTCGTGAGTACGAGCTTTCAGTAGCCCAGACGGTTT<br>TAAAGATTCACTCTCGTGTTGCCGACCTGTATAATGACCCCATGAACCAAA<br>TGGATCAGCAATTACGCTTAACTGTCGAGGCTTTGCGCGAACGCCAAGAA<br>CATGAGATGATCAACAACCGTGAATTTGGTTTACTGCATAATGCAGATTTAA<br>AGCAGCGCATCCATACGCGCTCCGGCCCCCCAACACCTGACGACCTGGA<br>CGAGTTAATCTCTCGCCGCCGCAAAACGCAAGTGTTGTTGGCGCATCCAC<br>GCACTATTGCCGCCATTGGTCGTGAGTGGAACGCGCGTGGAATCTATCCG<br>ACGGGCGCCGAATTGCATGGAACAGATGTACGCGCTTGGCGTGGAATTCC<br>TTTACTTCCTTGTAACAAAATTCCCGTAACCCCGGAACAAACAAGCAGTAT<br>CATTGCTATGCGCTTGGGTGAGGAAAACCAGGGGGTAGTCGGATTGCACC<br>AGACCGGCATTCCTGACGAGTATCAACCAGGCTTGTCTGTTCGTTTTATGG<br>GGATTAATGATCAGGCAGTCATCCAGTATCTTGTTAGCGCGTACTATTCAG<br>CAGCAGTGTTGGTGCCTGATGCTTTGGGGATTCTGGAGGATGTTGAAATC<br>GGGCATTGATTAACCTAGGCTGCTGC

Table S2. DNA sequences of PCR primers used to construct plasmids.

Primer	DNA Sequence
His-SI-Enc1-iPCR-FW	ATGCACCACCACCATCACCATACAATCGTAGACGAGACGTTAAATGGG
pETDuet-1-iPCR-RV	TGTATATCTCCTTCTTATACTTAACTAATATACTAAGATGGGG
His-SI-Enc2-iPCR-FW	ATGCACCACCACCATCACCATACTACGTCCGTGGATCCAACTTC
Double-Enc-His-SI-Enc2-iPCR-FW	ACTACGTCCGTGGATCCAACTTC
Double-Enc-His-SI-Enc2-iPCR-RV	ATGGTGATGGTGGTGGTGCATGTATATCTCCTTCTTATATCATAACCCG
Double-Enc-Strep-SI-Enc2-iPCR-RV	CTTTTCGAACTGCGGGTGGCTCCACATGTATATCTCCTTCTTATATCATAACCC
His-SI-Enc1-R195C-FW	TGTGCCGCCTTAGAAATCATCG
His-SI-Enc1-R195C-iPCR-RV	GCGCGGAAGCTCGACTAC
His-SI-Enc1-R195C-Gibson-Insert-RV	ACAGCGCGATAAAGTCAGTGC
His-SI-Enc1-R195C-Gibson-Vector-FW	ACTGACTTTATCGCGCTGTGATTATGAACAAGTCCTGG
His-SI-Enc1-R195C-Gibson-Vector-RV	GATTTCTAAGGCGGCACAGCGCGGAAGCTCGAC

Table S3. Primer pairs used to construct the plasmids used in this study.

Plasmid	Cloning Method	Primers
His-Sl-Enc1	Inverse PCR	His-Sl-Enc1-iPCR-FW<br>pETDuet-1-iPCR-RV
His-Sl-Enc2	Inverse PCR	His-Sl-Enc2-iPCR-FW<br>pETDuet-1-iPCR-RV
Sl-Enc1 / His-Sl-Enc2	Inverse PCR	Double-Enc-His-Sl-Enc2-iPCR-FW<br>Double-Enc-His-Sl-Enc2-iPCR-RV
His-Sl-Enc1 / Sl-Enc2	Inverse PCR	His-Sl-Enc1-iPCR-FW<br>pETDuet-1-iPCR-RV
His-Sl-Enc1 / His-Sl-Enc2	Inverse PCR	His-Sl-Enc1-iPCR-FW<br>pETDuet-1-iPCR-RV
His-Sl-Enc1 / Strep-Sl-Enc2	Inverse PCR	Double-Enc-His-Sl-Enc2-iPCR-FW<br>Double-Enc-Strep-Sl-Enc2-iPCR-RV
His-Sl-Enc1(R195C) / Strep-Sl-Enc2	Inverse PCR	His-Sl-Enc1-R195C-FW<br>His-Sl-Enc1-R195C-iPCR-RV
His-Sl-Enc1(R195C) / Strep-Sl-Enc2(G198C)	Gibson Assembly	Insert<br>His-Sl-Enc1-R195C-FW<br>His-Sl-Enc1-R195C-Gibson-Insert-RV
		Vector<br>His-Sl-Enc1-R195C-Gibson-Vector-FW<br>His-Sl-Enc1-R195C-Gibson-Vector-RV

Table S4. Protein sequences used in this work.

Protein	Protein Sequence
Sl-Enc1	MTIVDETLNGNPEDTAHSSLSTAAARNLATTTKTVPQMQGITSRWLLRL<br>LPWVQVSGGTYRVNRRLSHTVGDGRIDFDISGSDVAIIPEELRELPALR<br>DFTDTEVLAALGERFTQREYAPGELIAEAGRPGDRLVLIAHGRVDRIGT<br>GKYGDTTVLAALAGGDHLGDAPLTSDEVTWEFSYRAVTRVTVVELPRR<br>AALEIIERSPGLREHLAGVRQGPVHPTNSSGESAVAVASGHRGEPSLP<br>GAFADYDLAPREYELSVAQTVLRTHTRVGDLYNDPMNQVEEQLKLTVQ<br>ALRERQEHEMINNREFGLLHNADLKQRIPTRSGPPTPDDLDDLLATVW<br>KDPGFLLAHPRAIAAMAREWSARGLYPTAVDFHGHSLPSWRGVPIFPC<br>NKIPVTKERTSSILLLRTGEEKQGVVGLHQTGIPDEYEPSLSVRFMGIDD<br>RAVINYLVSAYYSAAVLVPDALGVLEDVEVGL*
His-Sl-Enc1	MHHHHHHTIVDETLNGNPEDTAHSSLSTAAARNLATTTKTVPQMQGIT<br>SRWLLRLLPWVQVSGGTYRVNRRLSHTVGDGRIDFDISGSDVAIIPEEL<br>RELPALRDFTDTEVLAALGERFTQREYAPGELIAEAGRPGDRLVLIAHG<br>RVDRIGTGKYGDTTVLAALAGGDHLGDAPLTSDEVTWEFSYRAVTRVT<br>VVELPRRAALEIIERSPGLREHLAGVRQGPVHPTNSSGESAVAVASGH<br>RGEPSLPGAFADYDLAPREYELSVAQTVLRTHTRVGDLYNDPMNQVE<br>EQLKLTVQALRERQEHEMINNREFGLLHNADLKQRIPTRSGPPTPDDL<br>DDLLATVWKDPGFLLAHPRAIAAMAREWSARGLYPTAVDFHGHSLPS<br>WRGVPIFPCNKIPVTKERTSSILLLRTGEEKQGVVGLHQTGIPDEYEPSL<br>SVRFMGIDDRAVINYLVSAYYSAAVLVPDALGVLEDVEVGL*
His-Sl-Enc1 (R195C)	MHHHHHHTIVDETLNGNPEDTAHSSLSTAAARNLATTTKTVPQMQGIT<br>SRWLLRLLPWVQVSGGTYRVNRRLSHTVGDGRIDFDISGSDVAIIPEEL<br>RELPALRDFTDTEVLAALGERFTQREYAPGELIAEAGRPGDRLVLIAHG<br>RVDRIGTGKYGDTTVLAALAGGDHLGDAPLTSDEVTWEFSYRAVTRVT<br>VVELPRCAALEIIERSPGLREHLAGVRQGPVHPTNSSGESAVAVASGH<br>RGEPSLPGAFADYDLAPREYELSVAQTVLRTHTRVGDLYNDPMNQVE<br>EQLKLTVQALRERQEHEMINNREFGLLHNADLKQRIPTRSGPPTPDDL<br>DDLLATVWKDPGFLLAHPRAIAAMAREWSARGLYPTAVDFHGHSLPS<br>WRGVPIFPCNKIPVTKERTSSILLLRTGEEKQGVVGLHQTGIPDEYEPSL<br>SVRFMGIDDRAVINYLVSAYYSAAVLVPDALGVLEDVEVGL*
Sl-Enc2	MTTSVDPTSGPQASGTEQNRSSLDTAAARKLATTTKTVPQMQGISSR<br>WLLRVLPWTQVNGGTYRVNRRLTHTLGDGQVEFVNTGAEVRVIPEEL<br>RELAPLRGFTDTPTLEALAGRFAQHEFAPGDVLAQQDQPADRIILIAHG<br>KLDRLGTGKYGGETVQGQLAGGDHLGAAALLDGGAAWEHTVRAVTR<br>VTALTLSRGDYEQVLGGSESLRAHVEAFRAALVPAQNKHGEAAIEVAA<br>GHVGEPSLPGTFADYDLAPREYELSVAQTVLKIHSRVADLYNDPMNQM<br>DQQLRLTVEALRERQEHEMINNREFGLLHNADLKQRIHTRSGPPTPDD<br>LDELISRRRKTQVLLAHPRTIAAIGREWNARGIYPTGAELHGTDVRAWR<br>GIPLLPCNKIPVTPEQTSSIIAMRLGEENQGVVGLHQTGIPDEYQPGLSV<br>RFMGINDQAVIQYLVSAYYSAAVLVPDALGILEDVEIGH*

His-Sl-Enc2	MHHHHHHTTSVDPTSGPQASGTEQNRSSLDTAAARKLATTTKTVPQMQGISSRWLLRVLPWTQVNGGTYRVNRRLTHTLGDGQVEFVNTGAEVRVIPEELRELAPLRGFTDTPTLEALAGRFAQHEFAPGDVLAQQDQPADRIILIAHGKLDRLGTGKYGGETVQGQLAGGDHLGAAALLDGGAAWEHTVRAVTRVTALTLSRGDYEQVLGGSESLRAHVEAFRAALVPAQNKHGEAAIEVAAGHVGEPSLPGTFADYDLAPREYELSVAQTVLKIHSRVADLYNDPMNQMDQQLRLTVEALRERQEHEMINNREFGLLHNADLKQRIHTRSGPPTPDDLDELISRRRKTQVLLAHPRTIAAIGREWNARGIYPTGAELHGTDVRAWRGIPLLPCNKIPVTPEQTSSIIAMRLGEENQGVVGLHQTGIPDEYQPGLSVRFMGINDQAVIQYLVSAYYSAAVLVPDALGILEDVEIGH*
Strep-Sl-Enc2	MWSHPQFEKTTSVDPTSGPQASGTEQNRSSLDTAAARKLATTTKTVPQMQGISSRWLLRVLPWTQVNGGTYRVNRRLTHTLGDGQVEFVNTGAEVRVIPEELRELAPLRGFTDTPTLEALAGRFAQHEFAPGDVLAQQDQPADRIILIAHGKLDRLGTGKYGGETVQGQLAGGDHLGAAALLDGGAAWEHTVRAVTRVTALTLSRGDYEQVLGGSESLRAHVEAFRAALVPAQNKHGEAAIEVAAGHVGEPSLPGTFADYDLAPREYELSVAQTVLKIHSRVADLYNDPMNQMDQQLRLTVEALRERQEHEMINNREFGLLHNADLKQRIHTRSGPPTPDDLDELISRRRKTQVLLAHPRTIAAIGREWNARGIYPTGAELHGTDVRAWRGIPLLPCNKIPVTPEQTSSIIAMRLGEENQGVVGLHQTGIPDEYQPGLSVRFMGINDQAVIQYLVSAYYSAAVLVPDALGILEDVEIGH*
Strep-Sl-Enc2 (G198C)	MWSHPQFEKTTSVDPTSGPQASGTEQNRSSLDTAAARKLATTTKTVPQMQGISSRWLLRVLPWTQVNGGTYRVNRRLTHTLGDGQVEFVNTGAEVRVIPEELRELAPLRGFTDTPTLEALAGRFAQHEFAPGDVLAQQDQPADRIILIAHGKLDRLGTGKYGGETVQGQLAGGDHLGAAALLDGGAAWEHTVRAVTRVTALTLSRCDYEQVLGGSESLRAHVEAFRAALVPAQNKHGEAAIEVAAGHVGEPSLPGTFADYDLAPREYELSVAQTVLKIHSRVADLYNDPMNQMDQQLRLTVEALRERQEHEMINNREFGLLHNADLKQRIHTRSGPPTPDDLDELISRRRKTQVLLAHPRTIAAIGREWNARGIYPTGAELHGTDVRAWRGIPLLPCNKIPVTPEQTSSIIAMRLGEENQGVVGLHQTGIPDEYQPGLSVRFMGINDQAVIQYLVSAYYSAAVLVPDALGILEDVEIGH*

Table S5. Cryo-EM data collection, refinement, and validation statistics.

	Sl-Enc1 (EMD-44632) (PDB ID: 9BJE)	Sl-Enc2 (EMD-44603) (PDB ID: 9BIX)
**Data collection and processing**		
Magnification	45,000x	105,000x
Voltage (kV)	200	300
Electron exposure (e–/Å^2)	45.50	54.0
Defocus range (μm)	-0.8 to -1.8	-0.5 to -1.0
Pixel size (Å)	0.91	0.84
Symmetry imposed	I	I
Initial particle images (no.)	77,425	288,651
Final particle images (no.)	69,590	155,190
Map resolution (Å)<br>FSC threshold	2.58<br>0.143	2.59<br>0.143
**Refinement**		
Initial model used (PDB code)	9BIX	9BHU
Model resolution (Å)<br>FSC threshold	2.8<br>0.5	2.7<br>0.5
Map sharpening *B* factor (Å^2)	-99.9	-114.4
Model composition<br>Non-hydrogen atoms<br>Protein residues<br>Ligands	 2,094<br>266<br>0	 1,944<br>244<br>0
B factors (Å^2)<br>Protein<br>Ligands	 39.30<br>-	 33.63<br>-
R.m.s. deviations<br>Bond lengths (Å)<br>Bond angles (°)	 0.005<br>1.112	 0.003<br>0.513
Validation<br>MolProbity score<br>Clashscore<br>Poor rotamers (%)	 1.41<br>4.53<br>0.88	 1.44<br>3.34<br>0.95
Ramachandran plot<br>Favored (%)<br>Allowed (%)<br>Disallowed (%)	 96.95<br>3.05<br>0	 95.42<br>4.58<br>0

References

[1] N. Oberg, R. Zallot, J. A. Gerlt, *J Mol Biol* **2023**, *435*, 168018.
[2] R. Zallot, N. Oberg, J. A. Gerlt, *Biochemistry* **2019**, *58*, 4169-4182.
[3] K. Katoh, J. Rozewicki, K. D. Yamada, *Brief Bioinform* **2019**, *20*, 1160-1166.
[4] I. Letunic, P. Bork, *Nucleic Acids Res* **2021**, *49*, W293-W296.
[5] C. Suloway, J. Pulokas, D. Fellmann, A. Cheng, F. Guerra, J. Quispe, S. Stagg, C. S. Potter, B. Carragher, *J Struct Biol* **2005**, *151*, 41-60.
[6] A. Punjani, J. L. Rubinstein, D. J. Fleet, M. A. Brubaker, *Nat Methods* **2017**, *14*, 290-296.
[7] E. F. Pettersen, T. D. Goddard, C. C. Huang, G. S. Couch, D. M. Greenblatt, E. C. Meng, T. E. Ferrin, *J Comput Chem* **2004**, *25*, 1605-1612.
[8] P. Emsley, K. Cowtan, *Acta Crystallogr D Biol Crystallogr* **2004**, *60*, 2126-2132.
[9] P. Emsley, B. Lohkamp, W. G. Scott, K. Cowtan, *Acta Crystallographica Section D: Biological Crystallography* **2010**, *66*, 486-501.
[10] P. D. Adams, P. V. Afonine, G. Bunkóczi, V. B. Chen, I. W. Davis, N. Echols, J. J. Headd, L. W. Hung, G. J. Kapral, R. W. Grosse-Kunstleve, A. J. McCoy, N. W. Moriarty, R. Oeffner, R. J. Read, D. C. Richardson, J. S. Richardson, T. C. Terwilliger, P. H. Zwart, *Acta Crystallographica Section D: Biological Crystallography* **2010**, *66*, 213-221.
[11] P. V. Afonine, B. P. Klaholz, N. W. Moriarty, B. K. Poon, O. V. Sobolev, T. C. Terwilliger, P. D. Adams, A. Urzhumtsev, *Acta Crystallogr D Struct Biol* **2018**, *74*, 814-840.
[12] D. Liebschner, P. V. Afonine, M. L. Baker, G. Bunkoczi, V. B. Chen, T. I. Croll, B. Hintze, L. W. Hung, S. Jain, A. J. McCoy, N. W. Moriarty, R. D. Oeffner, B. K. Poon, M. G. Prisant, R. J. Read, J. S. Richardson, D. C. Richardson, M. D. Sammito, O. V. Sobolev, D. H. Stockwell, T. C. Terwilliger, A. G. Urzhumtsev, L. L. Videau, C. J. Williams, P. D. Adams, *Acta Crystallogr D Struct Biol* **2019**, *75*, 861-877.
